# Self-Powered Flexible Multicolor Electrochromic Devices for Information Displays

**DOI:** 10.34133/research.0227

**Published:** 2023-09-14

**Authors:** Wenzhao Xue, Yun Zhang, Feng Liu, Yao Dou, Mei Yan, Wenshou Wang

**Affiliations:** School of Chemistry and Chemical Engineering, University of Jinan, Jinan 250022, P.R. China.

## Abstract

The development of self-powered flexible multicolor electrochromic (EC) systems that could switch different color without an external power supply has remained extremely challenging. Here, a new trilayer film structure for achieving self-powered flexible multicolor EC displays based on self-charging/discharging mechanism is proposed, which is simply assembled by sandwiching an ionic gel film between 2 cathodic nickel hexacyanoferrate (NiHCF) and Prussian blue (PB) nanoparticle films on indium tin oxide substrates. The display exhibits independent self-powered color switching of NiHCF and PB films with fast responsive time and high reversibility by selectively connecting the Al wire as anodes with the 2 EC films. Multicolor switching is thus achieved through a color overlay effect by superimposing the 2 EC films, including green, blue, yellow, and colorless. The bleaching/coloration process of the displays is driven by the discharging/self-charging mechanism for NiHCF and PB films, respectively, ensuring the self-powered color switching of the displays reversibly without an external power supply. It is further demonstrated that patterns can be easily created in the self-powered EC displays by the spray-coating method, allowing multicolor changing to convey specific information. Moreover, a self-powered ionic writing board is demonstrated based on the self-powered EC displays that can be repeatedly written freehand without the need of an external power source. We believe that the design concept may provide new insights into the development of self-powered flexible multicolor EC displays with self-recovered energy for widespread applications.

## Introduction

Smart chromogenic materials that can reversibly change color in response to external stimuli are of scientific and technological interest because of their enormous applications in optoelectronic, optical data recording, color display, sensors, and security systems [1–10]. Various external stimuli, including light, electricity, heat, magnetic field, and mechanical force, have been adopted to guide the reversible color switching of smart chromogenic materials [11–18]. Among them, self-powered electrochromic (EC) systems that possess the capability to reversibly switch color without an external power supply have recently aroused great interest owing to their potential applications in the next-generation electronic devices [19–26]. Recent pioneering works have successfully developed self-powered EC systems by using EC materials as cathodes and metal foils, such as Al, Zn, and Mg as anodes in aqueous electrolytes [26–30]. For instance, Wang et al. [31] reported a self-powered EC device in aqueous KCl electrolyte by using Prussian blue (KFe^2+^[Fe^3+^(CN)_6_], PB) and Al as cathodic EC film and anode, respectively. The PB/KCl/Al device shows the rapid bleaching process upon connecting the 2 electrodes and the slow coloration process via oxidation with oxygen or rapidly under external power source. We have recently demonstrated a self-powered quasi-solid-state EC device based on a bilayer film configuration by integrating the PB cathodic EC film with an ionic gel film [32]. The EC device exhibits excellent self-powered color switching and straightforward operation, which shows important advance in the research of self-powered EC systems and promotes their promising applications. These prototype results shed light on the promising future in self-powered EC systems; however, this field is still in its infancy and several unsolved problems exist, namely, (a) the EC materials developed in the self-powered EC systems have been mainly limited to PB, tungsten oxides, and polypyrrole, which significantly restricts their broad applications; (b) most reported self-powered EC systems are based on aqueous electrolytes, which need a complicated installation for operation and lack flexibility; (c) the self-powered EC color switching is usually limited to several cycles and the recovered process generally needs days in ambient and still relies on an external power source, restricting their applications; (d) multicolor switching has rarely been achieved in the self-powered EC systems owing to the lack of suitable EC materials and new design. All these limitations have become a big bottleneck for further smart applications of self-powered EC systems. Therefore, self-powered EC systems with long-awaited multicolor switching, energy self-recovery, easy controllability, high flexibility, and simple manufacturing process are highly desired. However, this remains a great challenge.

Here, we propose a new simplified trilayer film structure for achieving self-powered flexible EC displays with multicolor switching based on self-charged/discharged batteries. The trilayer structure is composed of a quasi-solid-state ionic gel film sandwiched between 2 EC cathodic films. As demonstrated in our previous study, an ionic polyacrylamide/lithium chloride (PAM/LiCl) gel film can be used as both quasi-solid-state electrolyte and ion storage medium [32]. The first challenge is to develop appropriate EC cathodic materials that can exhibit independent self-powered color switching behavior under the same working parameters. Since PB has excellent EC properties [33–35] and shows great potential in the self-powered EC system [23,31], we envisage that Prussian blue analogs (PBAs), such as nickel hexacyanoferrate (KNi^2+^[Fe^3+^(CN)_6_], NiHCF), are promising self-powered EC cathodic materials because PBAs have the similar face-centered cubic crystal structure and redox reaction with PB [36,37]. Moreover, previous study shows that PBAs with various color are easily attainable by changing the transition metals to coordinate with cyanide ligands [38,39], providing a potential library for designing self-powered multicolor switching system. In addition, color display applications of the self-powered EC systems generally require creating specific patterns in the EC films to convey information. Lithography platforms are feasible to create complex patterned EC devices; however, this approach requires predesigned photomasks and complex fabrication steps. We address this issue by introducing the spray-coating method for the rapid and simple fabrication of uniform NiHCF and PB nanoparticle films, which show excellent independent self-powered color switching properties between yellow and colorless, and blue and colorless, respectively, upon connecting and disconnecting the Al wire between the gel film and the cathodic EC films. Moreover, the self-powered color switching of the NiHCF/gel film and PB/gel film devices is driven by the discharging/self-charging mechanism, with an initial discharge capacity of 42 and 53.4 mA h g^−1^, respectively, which eliminates the reliance on an external power supply. As such, self-powered flexible EC systems with multicolor switching are achieved based on color overlay effect by superimposing the NiHCF and PB EC films in the trilayer film structure. Furthermore, because of the simple spray-coating method, self-powered multicolor EC displays with patterns are further demonstrated, which could exhibit multicolor changing to convey specific information. In addition, a self-powered ionic writing board is developed, which can be repeatedly written freehand without an external power supply using LiCl/PAM aqueous solution as ink. The self-powered flexible multicolor EC systems hold great promises in applications of displays, anti-counterfeiting, and wearable devices.

## Results and Discussion

**Fig. 1.**
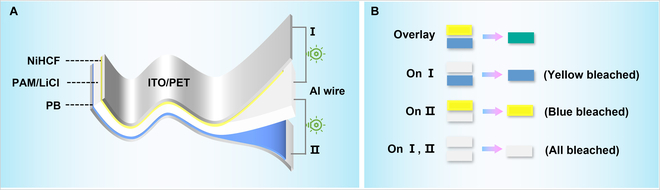
(A) Schematic illustration of the self-powered flexible multicolor display based on a trilayer film structure. (B) Schematic illustration of the self-powered multicolor display showing 4 colors obtained through the color overlay effect.

The design principle of the self-powered flexible multicolor EC display based on a trilayer film structure is schematically shown in Fig. 1A, which is composed of an ionic PAM/LiCl gel film sandwiched between NiHCF and PB nanoparticle films as 2 EC cathodes. Both the cathodic NiHCF and PB nanoparticle films exhibit independent self-powered color switching properties. Upon connecting the NiHCF nanoparticle film and the gel film with an Al wire, the yellow color of the NiHCF nanoparticle film rapidly bleaches to colorless due to reduction by electrons released from Al, corresponding to the discharging process of the NiHCF nanoparticle film/ionic gel film device, while the colorless reversibly switches back to its initial yellow state in ambient air after disconnecting the Al wire, accompanied by the self-charging process owing to the oxidation of reduced NiHCF. The PB nanoparticle film shows a similar self-powered color switching between blue and colorless accompanied by the discharging/self-charging process. As such, multicolor including green, blue, yellow, and colorless could be obtained by selectively connecting the Al wire with the 2 EC films through the intriguing color overlay effect (Fig. 1B).

Since the conductivity of NiHCF and PB nanoparticles is important for self-powered EC color switching, we develop a facile coprecipitation process to synthesize the nanoparticles without using any ligands. As shown in Fig. S1A, all the x-ray diffraction (XRD) peaks of PB nanoparticles are well indexed to PB and consistent with the reported PB [37,38,40]. The XRD peaks of NiHCF nanoparticles are similar to those of PB nanoparticles (Fig. S1A), indicating that NiHCF shares the same crystal structure with PB. The broad diffraction peaks of both NiHCF and PB nanoparticles indicate their small crystallite sizes of about 13.4 and 10.6 nm, respectively, as evaluated by the Scherrer equation [*D*_p_ = 0.94*λ*/*β*1/2 cos *θ*, where *D*_p_, *β*1/2, and *θ* represent the average crystallite size, line broadening in radians, and the Bragg angle, respectively] [36]. The TEM images further confirm the tiny size of NiHCF and PB nanoparticles (Fig. S1C and D). The small size of NiHCF and PB nanoparticles would benefit the spray-coating process for producing uniform nanoparticle films. The NiHCF and PB nanoparticles exhibit a strong absorption peak at 400 and 700 nm (Fig. S1B), consistent with their bright yellow and blue color of corresponding aqueous dispersions of nanoparticles (inset in Fig. S1C and D), respectively.

**Fig. 2.**
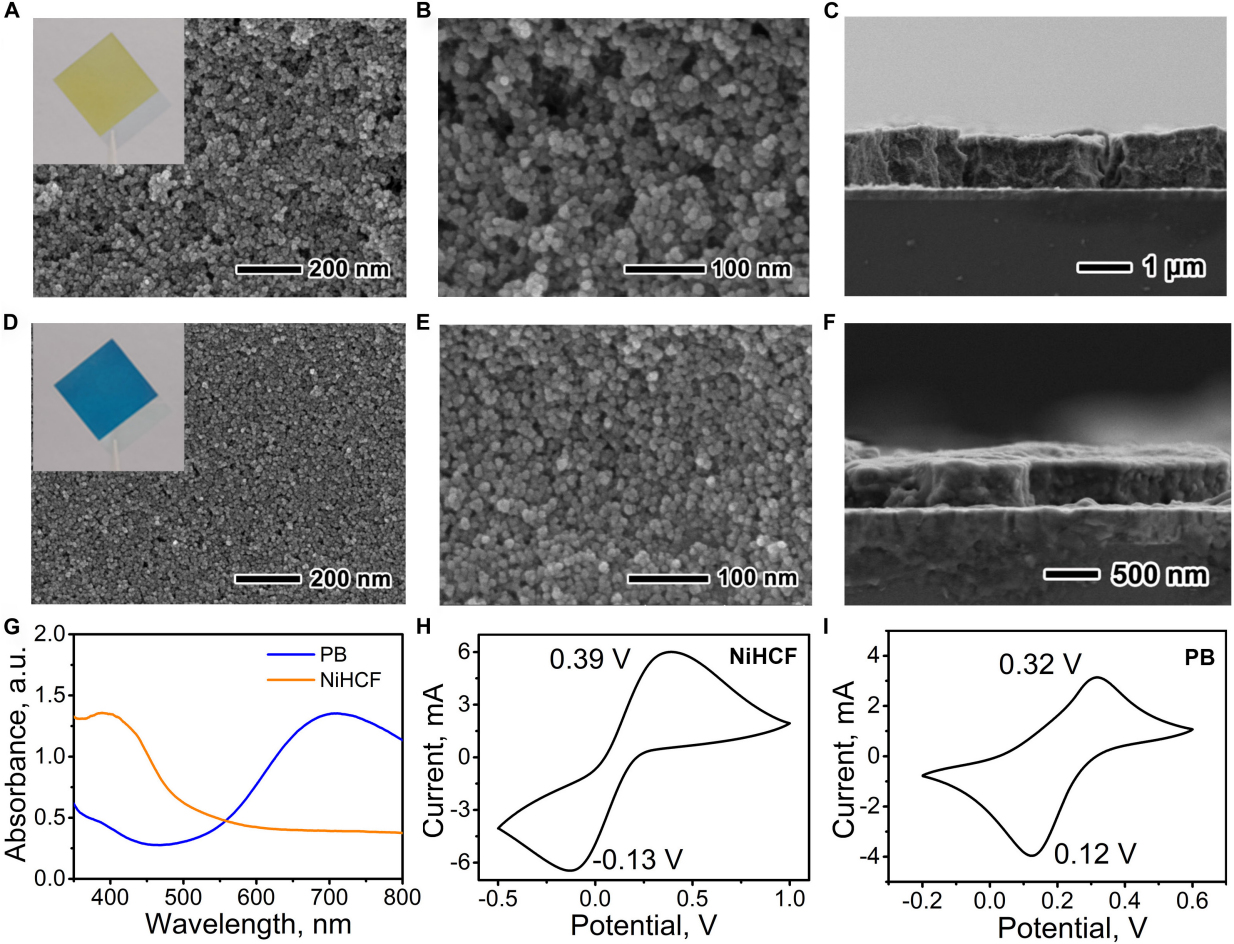
(A to F) SEM images of NiHCF (A to C) and PB (D to F) nanoparticle films from surface (A, B, D, and E) and cross-sectional (C and F) views. (G) UV-vis spectra of NiHCF and PB nanoparticle films. (H and I) Cyclic voltammograms of NiHCF (H) and PB (I) nanoparticle films at a scanning rate of 0.05 V s^−1^.

In order to improve the smoothness and conductivity of NiHCF and PB nanoparticle films, a small amount of poly(3,4-ethylenedioxythiophene):poly(styrenesulfonate) (PEDOT:PSS), as a typical high conductivity and transparent polymer, is introduced into the aqueous dispersions of nanoparticles for film preparation (Figs. S2 and S3). NiHCF and PB nanoparticles are easy to form nanoparticle films on indium tin oxide (ITO)/glass substrates with uniform yellow and blue color (inset in Fig. 2A and D, respectively). The low-magnification scanning electron microscopy (SEM) images show that both films have a uniform and flat surface (Fig. 2A to D). High-magnification SEM images indicate that both NiHCF and PB nanoparticles are homogeneously distributed on the ITO/glass without obvious aggregation, with an average diameter of 13 and 10 nm, respectively (Fig. 2B and E and Fig. S4). The cross-sectional SEM images indicate that the thickness of NiHCF and PB nanoparticle films is about 1.0 and 0.36 μm, respectively (Fig. 2C and F). The elemental mapping result indicates that C, N, Fe, K, and Ni coexist and distribute uniformly in the NiHCF nanoparticle film (Fig. S5), and C, N, Fe, and K coexist and distribute uniformly in the PB nanoparticle film (Fig. S6). Ultraviolet-visible (UV-vis) absorption spectra show that the NiHCF and PB nanoparticle films have an obvious absorption peak at 400 and 700 nm, respectively (Fig. 2G), consistent with their yellow and blue color. The cyclic voltammogram (CV) curve of the NiHCF nanoparticle film shows a redox pair at −0.13/0.39 V (Fig. 2H), which is attributed to the reduction and oxidation reaction of NiHCF [41,42]. The CV curve of the PB nanoparticle film exhibits a pair of peaks at 0.12/0.32 V (Fig. 2I), associated with the electrochemical switching between PB and colorless Prussian white (PW) [23,31].

**Fig. 3.**
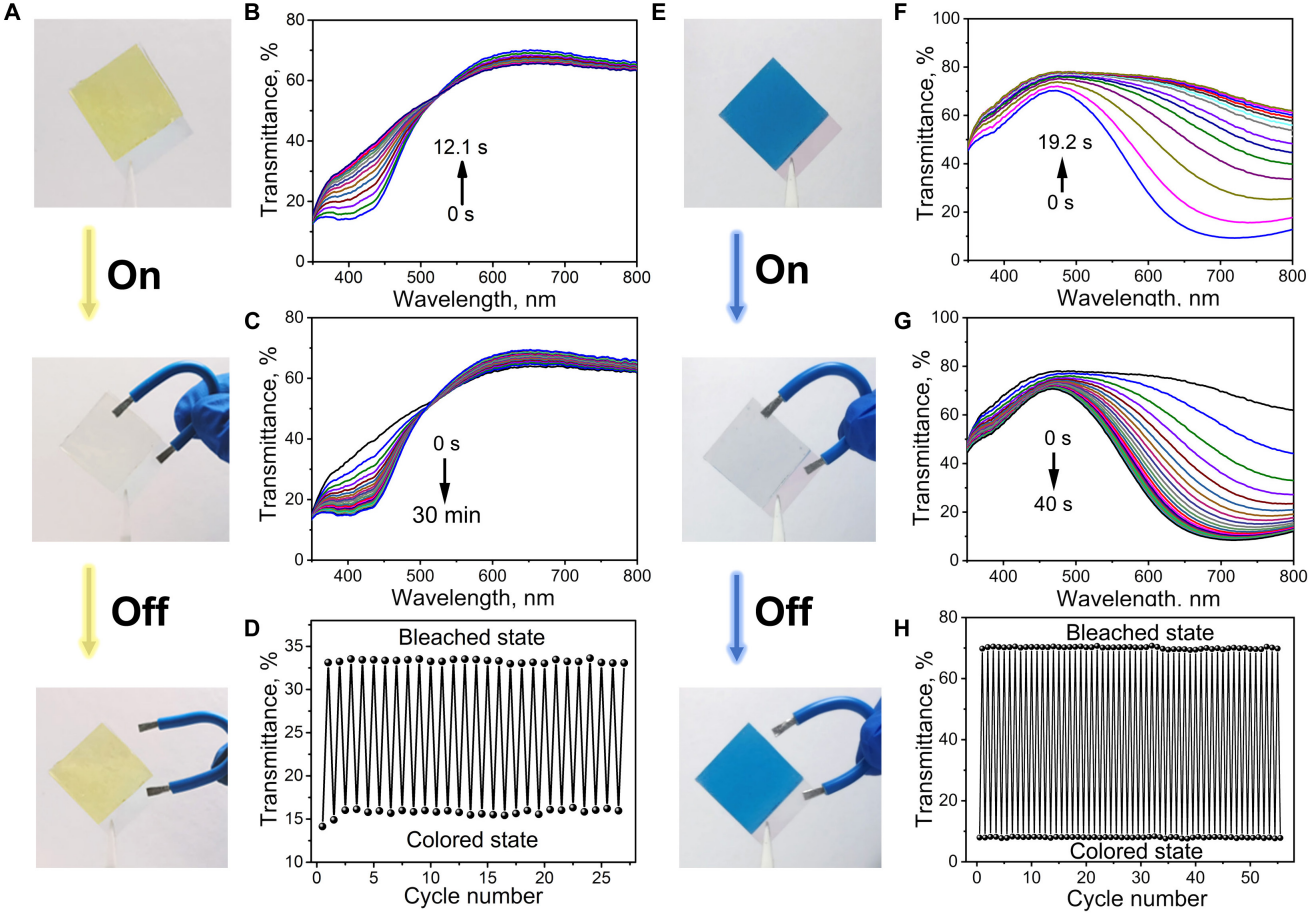
(A and E) Digital images showing the initial, bleached, and colored EC systems of NiHCF (A) and PB (E) nanoparticle films. (B, C, F, and G) UV-vis transmittance spectra showing the bleaching process upon connecting the Al wire with the NiHCF nanoparticle film (B) and the PB nanoparticle film (F), and the coloration process in air by disconnecting the Al wire with the NiHCF nanoparticle film (C) and the PB nanoparticle film (G). (D and H) Cycling properties of the EC systems of NiHCF (D) and PB (H).

The self-powered EC color switching properties of NiHCF and PB nanoparticle films are first evaluated based on a bilayer film configuration. The PAM/LiCl gel film with high transmittance and ionic conductivity acts as both quasi-solid-state electrolytes and ion storage medium (Fig. S7). As shown in Fig. 3A, self-powered EC system based on the NiHCF/gel film with initial yellow color is fabricated by simply attaching the ionic gel film on the NiHCF/ITO/glass film. The intensity of transmittance spectra at 400 nm increases gradually and saturates in 12.1 s upon connecting the NiHCF nanoparticle film and the gel film with an Al wire (Fig. 3B) because of the reduction of NiHCF. The UV-vis transmittance spectra of the colorless self-powered EC system reversibly switch back to its initial state in about 30 min in ambient air after disconnecting the Al wire (Fig. 3C). The bleaching and coloration time, Tb and Tc, is calculated as the time required to achieve 90% of final transmittance modulation [43,44], which is 8.5 s and 21.5 min, respectively (Fig. S8). In addition, the transmittance change of the self-powered EC system during the recovery process at 400 nm is 96% in the first 30 min and 4% for the next 90 min, respectively, showing a rapid coloration rate at the first 30 min. The reversibility and repeatability of the self-powered EC system is evaluated by monitoring the bleaching and coloration process repeatedly. Figure 3D shows the transmittance intensity at 400 nm of the EC system under yellow and colorless states within 25 cycles upon connecting and disconnecting the Al wire between the NiHCF nanoparticle film and the gel film, demonstrating good reversibility and repeatability in self-powered color switching.

Self-powered EC system based on the PB/gel film with blue color is then fabricated by attaching the gel film on the surface of the PB/ITO/glass film. The blue color of the self-powered EC system disappears rapidly upon connecting an Al wire between the PB nanoparticle film and the gel film and switches back reversibly upon disconnecting the Al wire between the 2 films, as shown in Fig. 3E. The time-dependent UV-vis transmittance spectra show that the intensity of transmittance at light wavelength 700 nm increases rapidly with time upon connecting the Al wire between the PB nanoparticle film and the gel film for 19.2 s (Fig. 3F). The transmittance spectra of colorless system rapidly recover in 40 s in ambient air after disconnecting the Al wire (Fig. 3G), which is much faster than the previous self-powered PB/KCl/Al device [31]. The bleaching and coloration time, Tb and Tc, is 18 and 29 s, respectively, for the self-powered EC system based on the PB/gel film (Fig. S9). Figure 3H shows the transmittance intensity at wavelength 700 nm of the self-powered EC system between blue and colorless states for 55 cycles by only connecting and disconnecting the Al wire between the PB nanoparticle film and the gel film, indicating high reversibility and repeatability in self-powered color switching without the need of external power source. In addition, the PB/gel film device with decreased amount of (NH_4_)_2_S_2_O_8_ (40 mg) also shows good self-powered color switching properties (Fig. S10). The bleaching and coloration time, Tb and Tc, is 6.4 s and 6.3 min, respectively, and the cycling number is 35, indicating that the amount of (NH_4_)_2_S_2_O_8_ could affect the color switching properties. Moreover, the self-powered color switching properties of the current EC system are comparable with the previous self-powered EC device by using the electrochemically deposited PB film while remaining other conditions the same [31,32]. These results demonstrate that spray coating nanoparticles is a promising approach for fabricating the EC film, especially for the PBA nanoparticle film, which could not be electrochemically deposited on ITO/glass substrates.

**Fig. 4.**
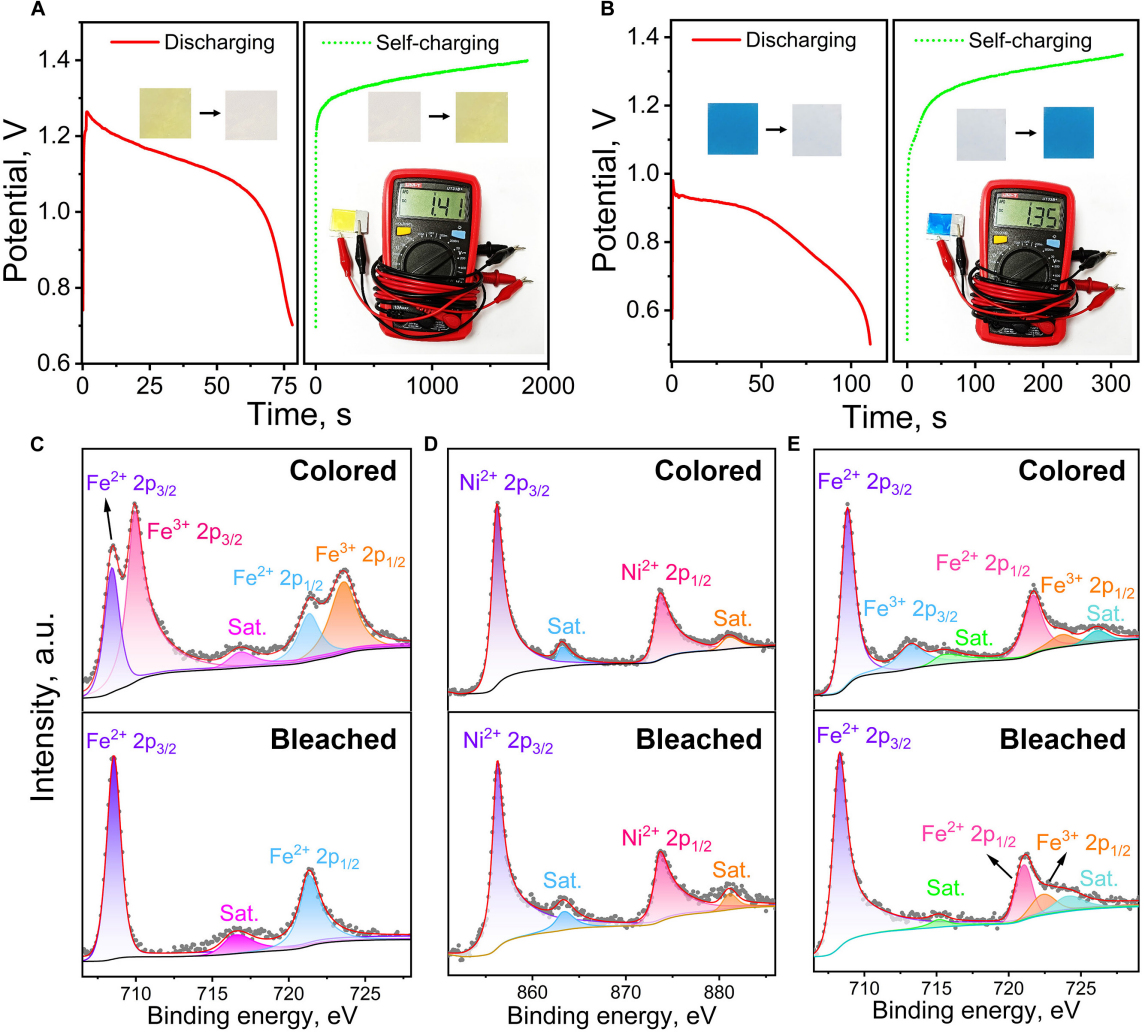
Discharging/self-charging behavior of the NiHCF/gel film device (A) and the PB/gel film device (B). The inset photos show the color change of the NiHCF/gel film device (A) and the PB/gel film device (B) and the corresponding OCP. (C and D) XPS spectra of Fe 2p region (C) and Ni 2p region (D) for the NiHCF nanoparticle film. (E) XPS spectra of Fe 2p region for the PB nanoparticle film.

To investigate the self-powered color switching mechanism, the bleaching and coloration processes of both the NiHCF/gel film and PB/gel film devices are first monitored by galvanostatic discharge curve and open circuit potential (OCP) measured in a 2-electrode configuration by using Al foil as both the counter and reference electrode. As shown in Fig. 4A, the NiHCF/gel film device decreases in potential with time at a current density of 2 A g^−1^ accompanied by the bleaching process of NiHCF from yellow to colorless, behaving as a discharging process of the device [45]. The OCP of the NiHCF/gel device increases to 1.41 V gradually after maintaining the colorless NiHCF/gel film device at air condition accompanied by the coloration process of NiHCF from colorless to yellow, acting as a self-charging process of the device [32]. The PB/gel device also decreases in potential with time at a current density of 2 A g^−1^ accompanied by the bleaching process from blue PB to colorless PW. The OCP of the PB/gel device gradually reaches 1.35 V after maintaining the colorless PB/gel film device at air condition accompanied by the coloration process from colorless PW to blue PB (Fig. 4B). The CV curves of the NiHCF/gel film and PB/gel film devices show a redox pair at 0.45/2.6 V and 0.5/1.9 V, respectively, which is related to the redox reactions of NiHCF and PB (Fig. S11). Therefore, the bleaching/coloration process of the NiHCF/gel film and PB/gel film devices is related to the discharging/self-charging process of the devices, respectively.

Subsequently, the NiHCF and PB nanoparticle films in the initial colored and bleached states are further studied by x-ray photoelectron spectroscopy (XPS) (Fig. 4C to E). The Fe 2p XPS spectrum of initial colored NiHCF exhibits 4 sharp peaks located at 708.4, 721.3, 709.9, and 723.5 eV (Fig. 4C). The peaks at 708.4 and 721.3 eV can be attributed to Fe^2+^ 2p_3/2_ and Fe^2+^ 2p_1/2_, and the peaks at 709.9 and 723.5 eV can be attributed to the presence of Fe^3+^ 2p_3/2_ and Fe^3+^ 2p_1/2_ [23,46–49]. After bleaching, the intensity of peaks related to Fe^2+^ 2p_3/2_ and Fe^2+^ 2p_1/2_ increases observably, while the peak related to Fe^3+^ 2p_3/2_ at 709.9 eV disappears. The change of Fe 2p XPS spectrum indicates that Fe^3+^ in NiHCF is reduced to Fe^2+^ by electrons released from the Al anode during the bleaching process. Moreover, the peaks of the Ni 2p XPS spectrum at 856.3 and 873.8 eV are attributed to Ni 2p_3/2_ and Ni 2p_1/2_ [37,41], respectively (Fig. 4D), and display no obvious variation for the colored and bleached NiHCF nanoparticle film, indicating that Ni is not involved in the color switching reaction. Similarly, for the PB nanoparticle film, the intensity of XPS peaks of Fe^3+^ at 712.6 and 723.5 eV decreases, while the peaks of Fe^2+^ at 708.4 and 721.1 eV increase in the bleached state, indicating that Fe^3+^ in PB is reduced to Fe^2+^ during the bleaching process (Fig. 4E).

**Fig. 5.**
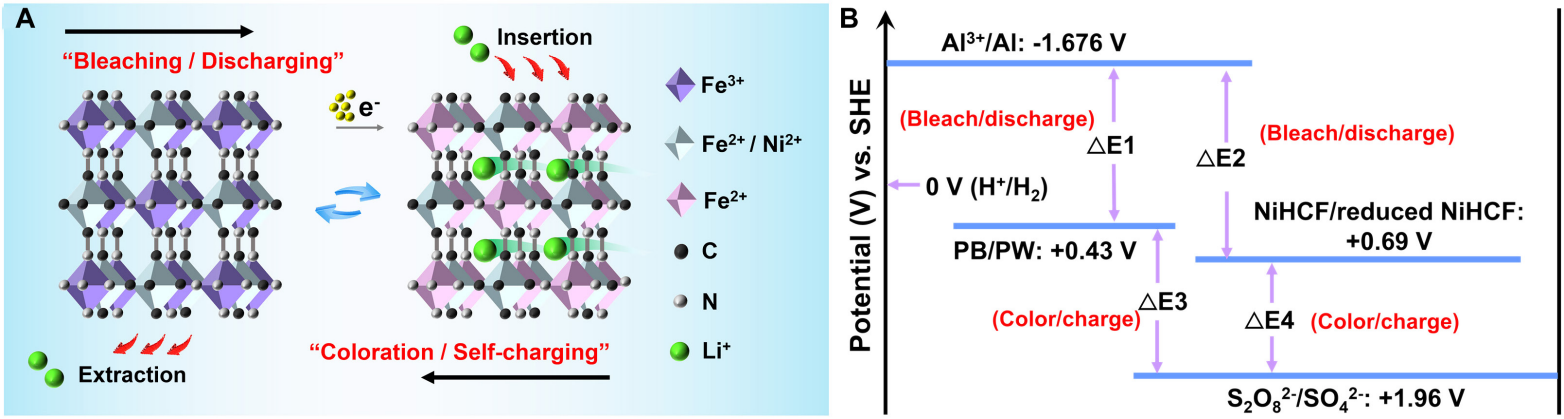
(A) Schematic illustration showing the self-powered color switching mechanism associated with the self-charging/discharging process. (B) Energy level transition diagram of the bleaching/discharging and coloration/charging processes of the self-powered flexible multicolor display.

Based on the above electrochemical analysis and XPS results, the self-powered color switching mechanism of NiHCF and PB is schematically shown in Fig. 5. Owing to the high redox potential of Al/Al^3+^ (−1.676 V versus Standard Hydrogen Electrode (SHE)), Al is used as an anode and could be easily oxidized to release electrons upon short circuiting the NiHCF or PB nanoparticle film with the gel film through an Al wire [31]. The released electrons would transfer to the EC films rapidly because of the large potential difference (NiHCF/reduced NiHCF, 0.69 V versus SHE; PB/PW, 0.43 V versus SHE) [23,28,42,50] and reduce KM^2+^[Fe^3+^(CN)_6_] to KLiM^2+^[Fe^2+^(CN)_6_] (M = Ni, Fe). Simultaneously, the reduction of NiHCF and PB is accompanied by the insertion of charge-balancing cations, such as Li^+^, derived from the PAM/LiCl gel film, into NiHCF and PB films (Reaction 1), resulting in the rapid color change from yellow to colorless and blue to colorless, respectively [32,40]. Moreover, the released electrons from Al to NiHCF or PB during the bleaching process give rise to power output for energy-related applications, behaving discharge characteristics of batteries with a galvanostatic discharge capacity of 42 and 53.4 mA h g^−1^ at a current density of 2 A g^−1^ for NiHCF and PB films, respectively (Fig. S12).

Since the redox potential of (NH_4_)_2_S_2_O_8_ (1.96 V versus SHE) is higher than that of oxygen (1.23 V versus SHE), the coloration process is mainly driven by (NH_4_)_2_S_2_O_8_ to oxidize the reduced NiHCF and PW [32]. As shown in Reaction 2, (NH_4_)_2_S_2_O_8_ in the gel film would act as a strong oxidant to accelerate the oxidation of KLiM^2+^[Fe^2+^(CN)_6_] to KM^2+^[Fe^3+^(CN)_6_] (M = Ni, Fe) accompanied by the extraction of Li^+^ cations from NiHCF and PB host into the gel film based on Reaction 2 upon disconnecting the Al wire between EC films and gel film. Moreover, increasing the amount of (NH_4_)_2_S_2_O_8_ in the self-powered EC displays could significantly enhance the coloration rate and improve the cycling performance (Fig. 3E to H and Fig. S10), further demonstrating that high amount of (NH_4_)_2_S_2_O_8_ could accelerate the oxidation kinetics to oxidize the reduced KLiM^2+^[Fe^2+^(CN)_6_] to KM^2+^[Fe^3+^(CN)_6_] (M = Ni, Fe) based on Reaction 2. Since (NH_4_)_2_S_2_O_8_ is gradually consumed during the coloration process, a high amount of (NH_4_)_2_S_2_O_8_ in the self-powered ECDs could elongate the cycling performance. During the coloration process, the NiHCF/gel film and PB/gel film devices spontaneously recover to their initial states (charged), which could be considered as a self-charging process [23,45]. The spontaneous self-charging of the devices ensures their cycling use for self-powered color switching, which eliminates the reliance on an external power source and significantly benefits its real applications.

Reaction 1 (Bleaching/discharging process):

3KM^2+^[Fe^3+^(CN)_6_] (colored) + 3Li^+^ + Al = 3KLiM^2+^[Fe^2+^(CN)_6_] (colorless) + Al^3+^

Reaction 2 (Coloration/self-charging process):

2KLiM^2+^[Fe^2+^(CN)_6_] (colorless) + S_2_O_8_^2−^ = 2KM^2+^[Fe^3+^(CN)_6_] (colored) + 2Li^+^ + 2SO_4_^2−^

M = Ni, Fe

**Fig. 6.**
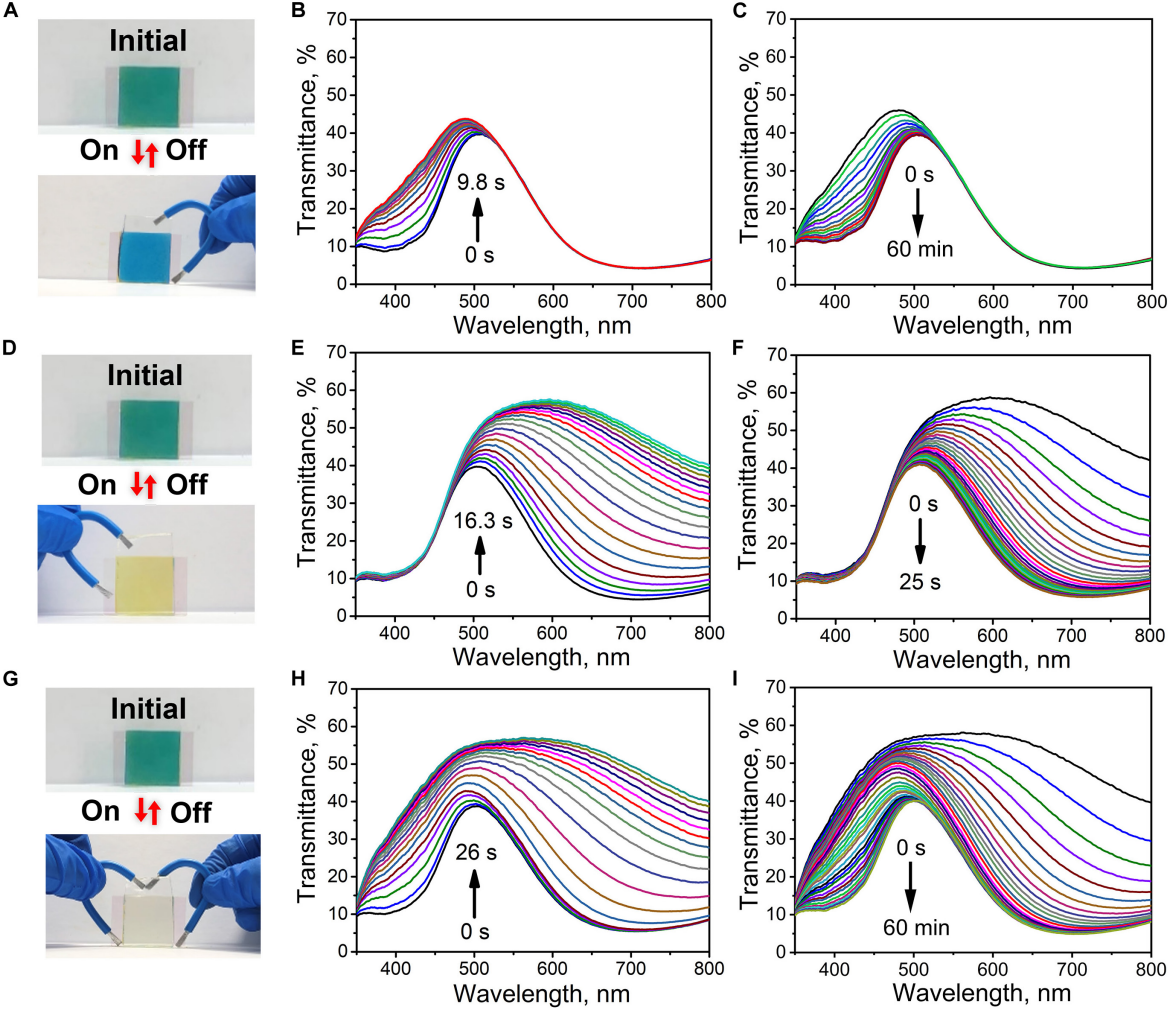
(A, D, and G) Digital photographs of self-powered multicolor EC system with different connection modes. (B and C) UV-vis transmittance spectra showing the bleaching (B) and coloration (C) processes upon connecting and disconnecting the Al wire between the NiHCF film and the PAM/LiCl gel film. (E and F) UV-vis transmittance spectra showing the bleaching (E) and coloration (F) processes upon connecting and disconnecting the Al wire between the PB film and the PAM/LiCl gel film. (H and I) UV-vis transmittance spectra showing the bleaching (H) and coloration (I) processes upon both connecting and disconnecting the Al wire between the PB film, the NiHCF film, and the PAM/LiCl gel film.

On the other hand, the NiHCF nanoparticle film and the PB nanoparticle film are easily attached on both the top and bottom surfaces of the PAM/LiCl gel film, respectively, to form a NiHCF/gel film/PB trilayer structure. The initial EC system exhibits a green color owing to color overlay of the top yellow EC film (NiHCF) and bottom blue EC film (PB) (Fig. 6). The transmittance spectrum of the EC system shows combined spectra of NiHCF and PB nanoparticle films. By connecting an Al wire between the NiHCF nanoparticle film and the gel film, the NiHCF nanoparticle film changes to colorless, while the PB nanoparticle film is in the initial blue state, generating a blue color (Fig. 6A). The intensity of transmittance at light wavelength 400 nm increases gradually owing to the reduction of KNi^2+^[Fe^3+^(CN)_6_] to KLiNi^2+^[Fe^2+^(CN)_6_], while the intensity of transmittance at light wavelength 700 nm does not change obviously (Fig.6B and C). Upon connecting the PB nanoparticle film and the gel film with an Al wire, the PB nanoparticle film changes to colorless while the NiHCF nanoparticle film is in the initial yellow state, thus presenting a yellow color (Fig. 6D). The corresponding time-dependent transmittance spectra indicate that the intensity of transmittance at light wavelength 700 nm that belonged to the PB nanoparticle film increases gradually in a short period of 16.3 s owing to the reduction of PB to PW, while the intensity of transmittance at light wavelength 400 nm that belonged to the NiHCF nanoparticle film does not change obviously (Fig. 6E and F). When both the NiHCF nanoparticle film and the PB nanoparticle film are connected with the gel film by the Al wire, the EC system exhibits colorless because both the films change to colorless (Fig. 6G). Accordingly, the intensity of transmittance at wavelength of 400 and 700 nm increases gradually with time, consistent with the color changing of the self-powered EC system (Fig.6H and I). Compared with previously reported results, the current systems show great advantages of multicolor, self-powered, self-recovered, fast response, and high reversibility (Table S1).

**Fig. 7.**
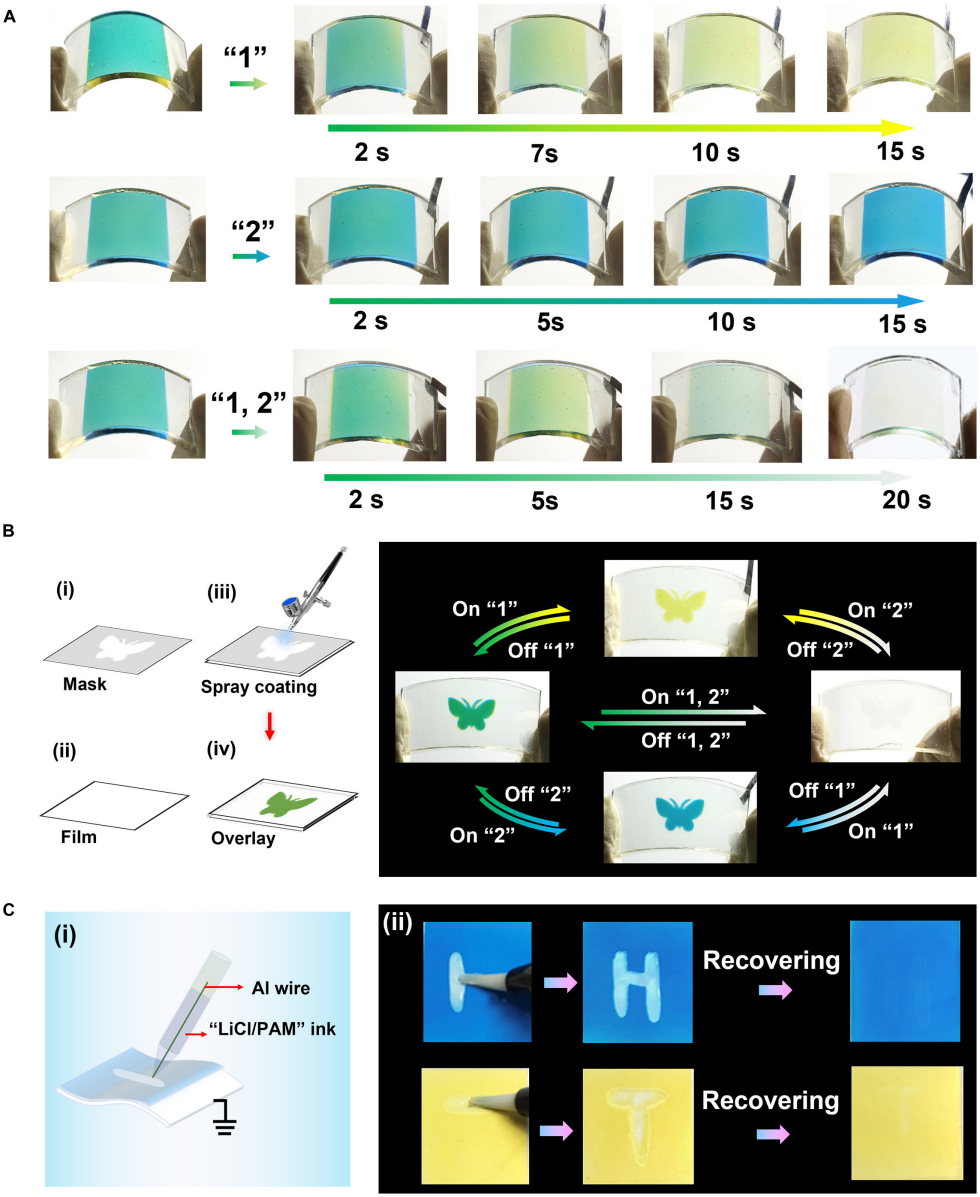
(A) Digital photographs of the self-powered flexible multicolor EC system showing different color changing upon different connection sequence. (B) Schematic fabrication process and digital photographs of the patterned self-powered multicolor EC system. (C) Schematic diagram of the self-powered ionic writing board (i) and the digital photographs showing the writing and recovering process (ii).

Self-powered flexible multicolor EC display is further developed by using ITO/polyethylene terephthalate (PET) as substrates, as shown in Fig. 7A. When the PB nanoparticle film is connected with the gel film by an Al wire, the flexible self-powered EC display exhibits a gradual green-to-yellow color change because of the color bleaching of blue PB to colorless PW. The display could gradually switch color to initial green after disconnecting the Al wire owing to the oxidation of colorless PW to blue PB. Similarly, the display changes color gradually from green to blue when the NiHCF nanoparticle film is connected with the gel film by an Al wire due to the bleaching of yellow KNi^2+^[Fe^3+^(CN)_6_] to colorless KLiNi^2+^[Fe^2+^(CN)_6_]. Upon connecting the gel film with PB and NiHCF nanoparticle films by the Al wire simultaneously, the display changes color gradually from green to colorless. The self-powered multicolor EC system with patterns can be simply fabricated by using spray-coated homogeneous solutions, which holds great promise for the applications in display and anti-counterfeiting. As a proof of concept, a self-powered EC display with patterns that exhibit a remarkable multicolor change is prepared (Fig. 7B and Fig. S13). Furthermore, we demonstrate a self-powered ionic writing board based on the self-powered EC system without any external energy supply (Fig. 7C). When the pen brush short-connects with the PB or NiHCF nanoparticle films by the Al wire, the potential differences between the Al and PB or NiHCF spontaneously drive self-bleaching to achieve the writing function. The information of “H” and “T” are written on the writing board based on PB and NiHCF nanoparticle films, respectively, because the contacted areas with pen are reduced by electrons released from Al wires. The information could be restored to the coloring state in the air after writing, which indicates that the writing board has excellent presentation without external power consumption. Considering the advantages of multicolor switching, straightforward operation, simple fabrication process, and flexibility, the self-powered EC device with patterns promises great potential toward its practical applications in the field of color display, anti-counterfeiting, and stored energy.

## Conclusion

In summary, we have developed a new self-powered flexible EC display with multicolor switching based on a trilayer film structure driven by discharging/self-charging mechanism. The trilayer film structure is simply assembled by sandwiching an ionic PAM/LiCl gel film between 2 cathodic NiHCF and PB nanoparticle films on ITO substrates prepared by a spray-coating method. The NiHCF and PB nanoparticle films show excellent self-powered color switching properties between blue and colorless, and yellow and colorless, upon connecting and disconnecting the Al wire as anodes with the 2 EC films, respectively. The bleaching process of the self-powered EC display is due to the reduction of NiHCF and PB by electrons released from the Al anode, which simultaneously behaves discharge characteristics of batteries for NiHCF and PB films, respectively. For the coloration process, the discharged self-powered EC displays could rapidly recover to their charged states through the oxidation reaction by (NH_4_)_2_S_2_O_8_ in the ionic gel film, which is a self-charging process. The self-charging of the EC displays ensures their cycling use for self-powered color switching without the need of any external power sources. A color overlay effect is then achieved to generate multicolor switching by superimposing the 2 cathodic PB and NiHCF nanoparticle films in the self-powered EC system. The current system exhibits great advances in multicolor switching among green, blue, yellow, and colorless, fast response time, high reversibility, straightforward operation, simple fabrication process, and high flexibility. Considering these advantageous features, the self-powered flexible multicolor EC system with patterns that possess dynamic color changing to convey specific information is developed for potential applications in high-level anti-counterfeiting. Moreover, we have demonstrated the self-powered ionic writing board based on the self-powered EC devices that can be repeatedly written freehand using LiCl/PAM aqueous solution as ink. Our result represents a new strategy for designing self-powered flexible multicolor EC system and significantly broadens their applications.

## Materials and Methods

### Materials

FeCl_2_·4H_2_O, NiCl_2_·6H_2_O, K_3_[Fe(CN)_6_], (NH_4_)_2_S_2_O_8_, and LiCl·H_2_O were purchased from Sinopharm Chemical Reagent Co. Ltd. Acrylamide (AAm) and *N*,*N*-methylenebisacrylamide (MBAA) were purchased from Sigma-Aldrich. Tetramethyl-ethylenediamine (TEMED) and aluminum (Al) were purchased from Macklin Biochemical Co. Ltd. PEDOT:PSS was purchased from Sigma-Aldrich. All chemicals were of analytical grade and used without any further purification. Flexible ITO/PET films were provided by Lelin Technology (Shenzhen) Ltd. The ITO/glass was purchased from Luoyang Nuozhuo Technology Co. Ltd.

### Synthesis of NiHCF and PB nanoparticles

In a typical synthesis of NiHCF nanoparticles, K_3_[Fe(CN)_6_] (2 mmol) and NiCl_2_·6H_2_O (2 mmol) were dissolved in distilled water (100 ml), respectively. Then, aqueous K_3_[Fe(CN)_6_] solution was added to aqueous NiCl_2_ solution slowly at room temperature under magnetic stirring. After reaction for 6 h, yellow precipitates were obtained upon adding acetone and centrifuging at 11,000 rpm for 10 min. The precipitate above was dispersed into deionized water (20 ml). PB nanoparticles were synthesized by the similar procedure by using FeCl_2_·4H_2_O instead of NiCl_2_·6H_2_O.

### Preparation of NiHCF and PB nanoparticle films

ITO/glass was sequentially washed with deionized water, acetone, ethanol, and deionized water in an ultrasonic bath each for 15 min. ITO/PET was used directly. A suspension of NiHCF or PB nanoparticles [mixed solvents of ethanol and deionized water, 2:1 (v/v)] was sprayed on the cleaned ITO/glass or ITO/PET at 50 °C to form NiHCF and PB nanoparticle films, respectively. The mass of NiHCF and PB nanoparticles on the ITO/glass was about 1.0 and 0.4 mg, respectively.

### Preparation of the ionic PAM/LiCl gel film

PAM/LiCl gel was prepared by our previously reported approach with slight modifications [32]. Typically, a homogeneous solution containing H_2_O (15 ml), LiCl (63 mmol), AAm (5.0 g), MBAA (20 mg), (NH_4_)_2_S_2_O_8_ (400 mg), and TEMED (5 μl) was well mixed and transferred to a plastic mold, followed by heating at 40 °C for 20 min. Then, the PAM/LiCl gel film was cut into rectangles for experiments.

### Fabrication of the self-powered NiHCF/gel film and PB/gel film devices

The self-powered NiHCF/gel film device was fabricated with a bilayer configuration, in which an ionic PAM/LiCl gel film was simply attached on the top of the NiHCF nanoparticle film. The bleaching and coloration processes of the device were achieved by connecting or disconnecting the Al wire between the NiHCF nanoparticle film and the gel film, respectively. The PB/gel film device was fabricated by the similar procedure using the PB nanoparticle film.

### Fabrication of the self-powered flexible multicolor EC displays

The self-powered flexible multicolor EC displays were fabricated with a trilayer configuration, by sandwiching an ionic PAM/LiCl gel film between the NiHCF nanoparticle film and the PB nanoparticle film on the ITO/PET substrate. The bleaching and coloration processes of the display were achieved by connecting or disconnecting a strip of the Al wire between NiHCF nanoparticle and/or PB nanoparticle films and the ionic PAM/LiCl gel film.

### Fabrication of the self-powered ionic writing board

The NiHCF nanoparticle film and the PB nanoparticle film were acted as the self-powered ionic writing board. The “LiCl/PAM” ink was prepared by dissolving LiCl (63 mmol) and (NH_4_)_2_S_2_O_8_ (40 mg) in H_2_O (15 ml). The prepared ink was packed into a pen refill, and a thin Al wire (with a diameter of 0.1 mm) was inserted into the ink in the brush body.

### Characterization

Powder XRD pattern was supported on a Rigaku D/Max 2200pc diffractometer equipped with graphite monochromatized Cu Kα radiation (*λ* = 0.15418 nm). Transmission electron microscopy (TEM) images were collected with a Hitachi-7700 microscope. SEM images were obtained using a Hitachi SU8010 microscope and Zeiss Gemini300. The UV-vis transmittance spectra were recorded using a UV-vis spectrometer (DH-2000-BAL, Ocean Insight). XPS characterization was conducted on an XPS system (ESCALAB Xi+) equipped with Al Kα radiation. Electrochemical measurements were carried out using a CHI 760 electrochemical workstation. The working electrodes were prepared by spray-coating the NiHCF and PB nanoparticles on an ITO glass, respectively. Al foil was used as the counter electrode and reference electrode. The AC impedance measurement was carried out in the water at room temperature. The working electrode was prepared by drop-coating the PB nanoparticles on a glassy carbon electrode. Pt was used as the counter electrode, and saturated Ag/AgCl was used as the reference electrode. The optical microscope images were recorded on a MIT500 microscope. The digital photographs were captured using a cellphone.

## Data Availability

All relevant data that support the findings are available within this article and supporting information and are also available from authors upon reasonable request.
