# Supplementary material for: Self-Powered Flexible Multicolor Electrochromic Devices for Information Displays
Source: Research (Wash D C). 2023 Sep 14;6:0227. doi: 10.34133/research.0227 (PMC10501365; doi:10.34133/research.0227)
Supplement: Supplementary 1 — Figs. S1 to S13 Table S1 [file research.0227.f1.docx]

**Supplementary Materials**

**Self-powered flexible multicolor electrochromic devices for information displays**

Wenzhao Xue, Yun Zhang, Feng Liu, Yao Dou, Mei Yan, Wenshou Wang*

*School of Chemistry and Chemical Engineering, University of Jinan, Jinan 250022, P.R. China*

**Corresponding Author: E-mail address: wangws@ujn.edu.cn.*

**Table of Contents:**

**Figure S1.** (a) XRD patterns of NiHCF and PB nanoparticles. (b) UV-vis spectra of NiHCF and PB nanoparticles. (c, d) TEM images of NiHCF (c) and PB (d) nanoparticles. Insets show the color of corresponding aqueous solutions of NiHCF and PB nanoparticles.

**Figure S2.** (a, b) The optical microscopy images of PB nanoparticle film with PEDOT: PSS (a) and without PEDOT: PSS (b).

**Figure S3.** AC impedance spectra of the PB nanoparticles with and without PEDOT: PSS.

**Figure S4.** (a, c) SEM images of the NiHCF (a) and PB (c) nanoparticles, and (b, d) the corresponding size distribution histograms of the NiHCF (b) and PB (d) nanoparticles.

**Figure S5.** (a) The typical SEM image of the NiHCF nanoparticle film and (b-f) the corresponding elemental distribution of C (b), N (c), Fe (d), K (e) and Ni (f).

**Figure S6.** (a) The typical SEM image of the PB nanoparticle film and (b-e) the corresponding elemental distribution of C (b), N (c), Fe (d) and K (e).

**Figure S7.** (a) Digital image of the PAM/LiCl gel film. (b) SEM image of the freeze-dried gel. (c) AC impedance spectra of the PAM/LiCl gel film.

**Figure S8.** (a, b) Change of light transmittance at a wavelength of 400 nm of the NiHCF/gel film device versus time during bleaching (a) and coloration (b) processes.

**Figure S9.** (a, b) Change of light transmittance at a wavelength of 700 nm of the PB/gel film device versus time during bleaching (a) and coloration (b) processes.

**Figure S10.** (a, b) UV-vis transmittance spectra showing bleaching process of PB/gel film device with 40 mg of (NH_4_)_2_S_2_O_8_ in the gel upon connecting the Al wire with PB nanoparticle film (a), and coloration process in air by disconnecting Al wire with PB nanoparticle film (b), respectively. (c) Change of light transmittance at a wavelength of 700 nm of the PB/gel film device versus time during bleaching (a) and coloration (b) processes. (d) The cycling property of the PB/gel film device.

**Figure S11.** (a, b) The cyclic voltammogram of NiHCF/gel film device (a) and PB/gel film device (b) at a scan rate of 0.05 V s^-1^.

**Figure S12.** (a, b) Galvanostatic discharge curves of the NiHCF/gel film device (a) and PB/gel film device (b) at a current density of 2 A g^-1^. (c) Digital images of the NiHCF/gel film and PB/gel film acted as self-rechargeable batteries.

**Figure S13.** Digital images of the self-powered multicolor EC display with patterns showing color changing upon different connection sequence.

**Table S1**. Comparison of our self-powered multicolor EC display with the electrochemical devices reported before.


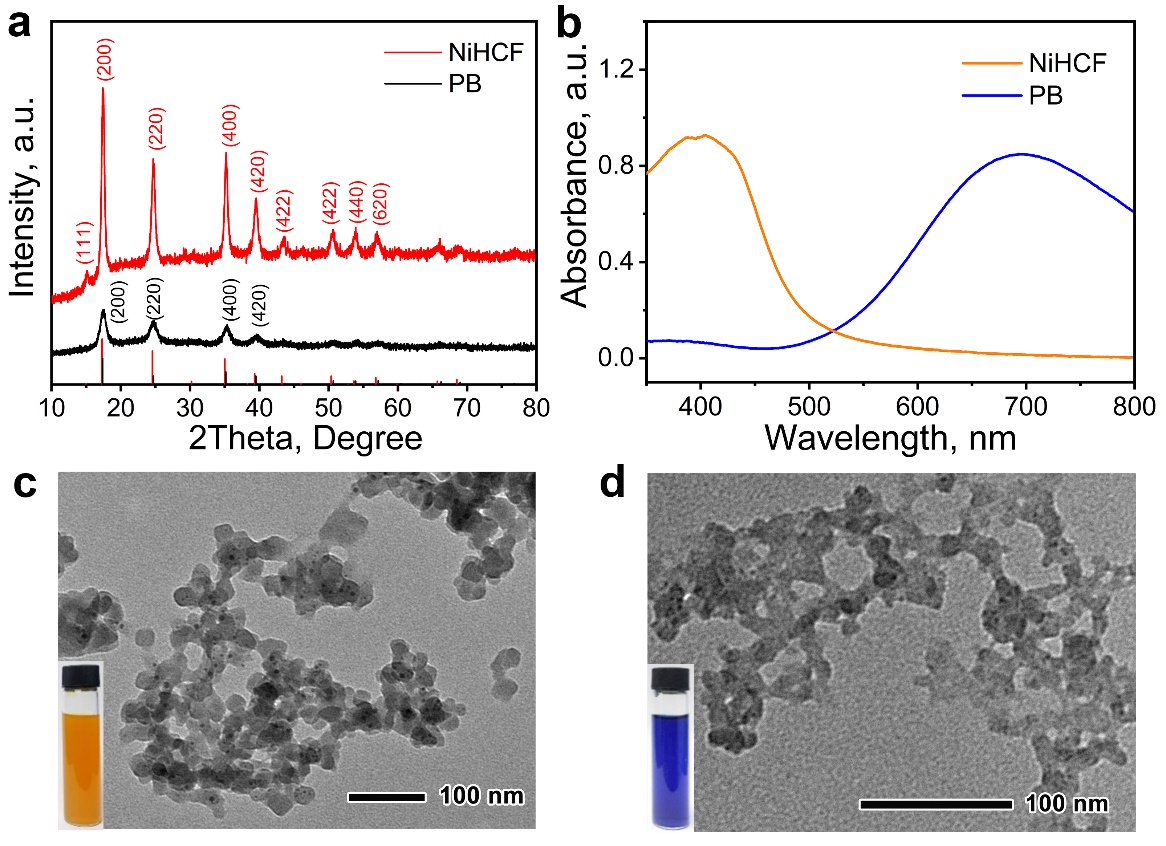


**Figure S1**. (a) XRD patterns of NiHCF and PB nanoparticles. (b) UV-vis spectra of NiHCF and PB nanoparticles. (c, d) TEM images of NiHCF (c) and PB (d) nanoparticles. Insets show the color of corresponding aqueous solutions of NiHCF and PB nanoparticles.


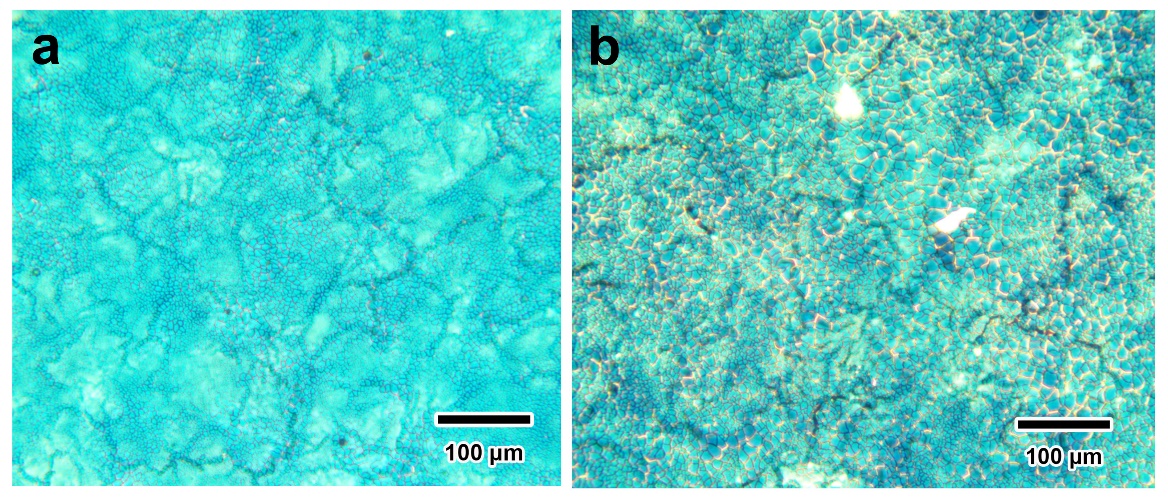


**Figure S2**. (a, b) The optical microscopy images of PB nanoparticle film with PEDOT: PSS (a) and without PEDOT: PSS (b).

The addition of PEDOT: PSS could improve the smoothness and decrease microscale cracks of the PB nanoparticle films.


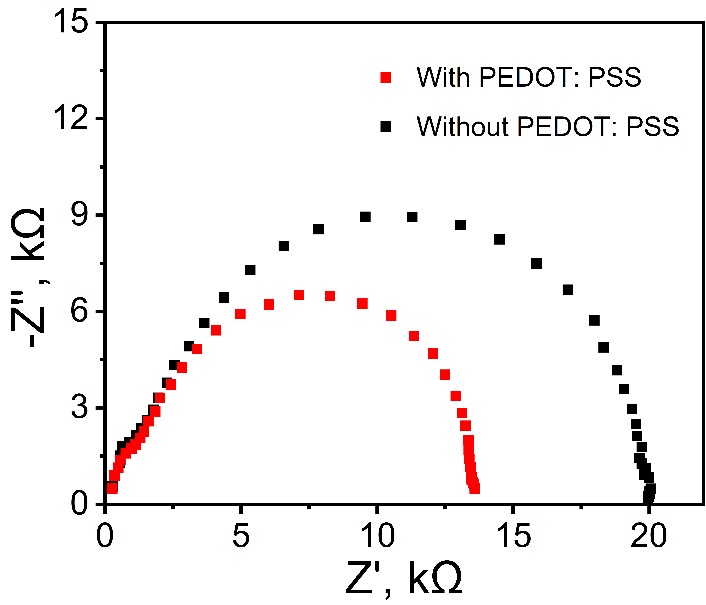


**Figure S3.** AC impedance spectra of the PB nanoparticles with and without PEDOT: PSS.

The PB nanoparticle film with PEDOT:PSS has smaller arc radius than that of film without PEDOT:PSS, indicating that the addition of PEDOT:PSS reduces interfacial resistance.


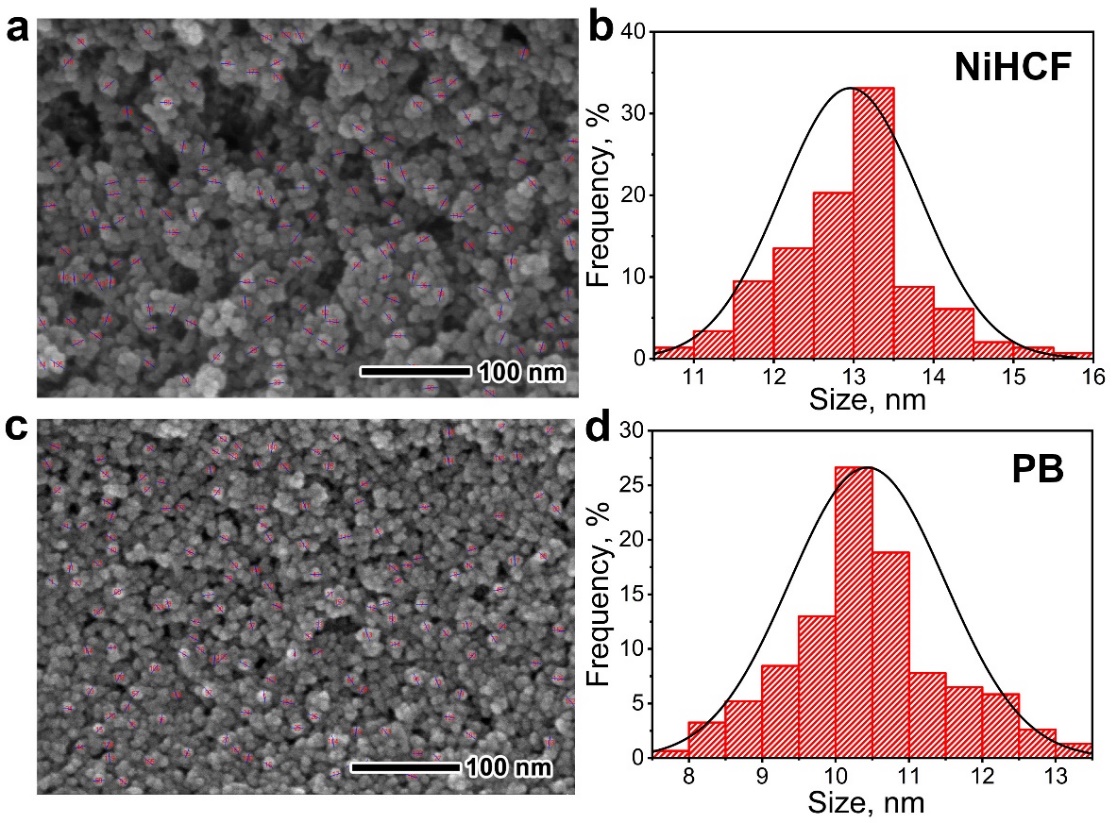


**Figure S4**. (a, c) SEM images of the NiHCF (a) and PB (c) nanoparticles, and (b, d) the corresponding size distribution histograms of the NiHCF (b) and PB (d) nanoparticles.


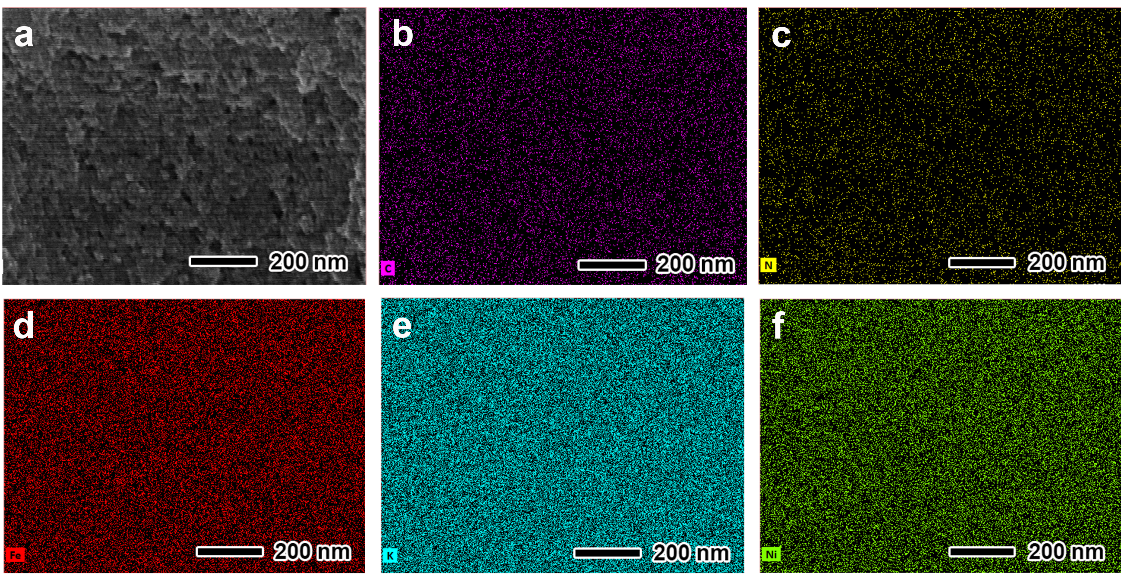


**Figure S5**. (a) The typical SEM image of the NiHCF nanoparticle film and (b-f) the corresponding elemental distribution of C (b), N (c), Fe (d), K (e) and Ni (f).


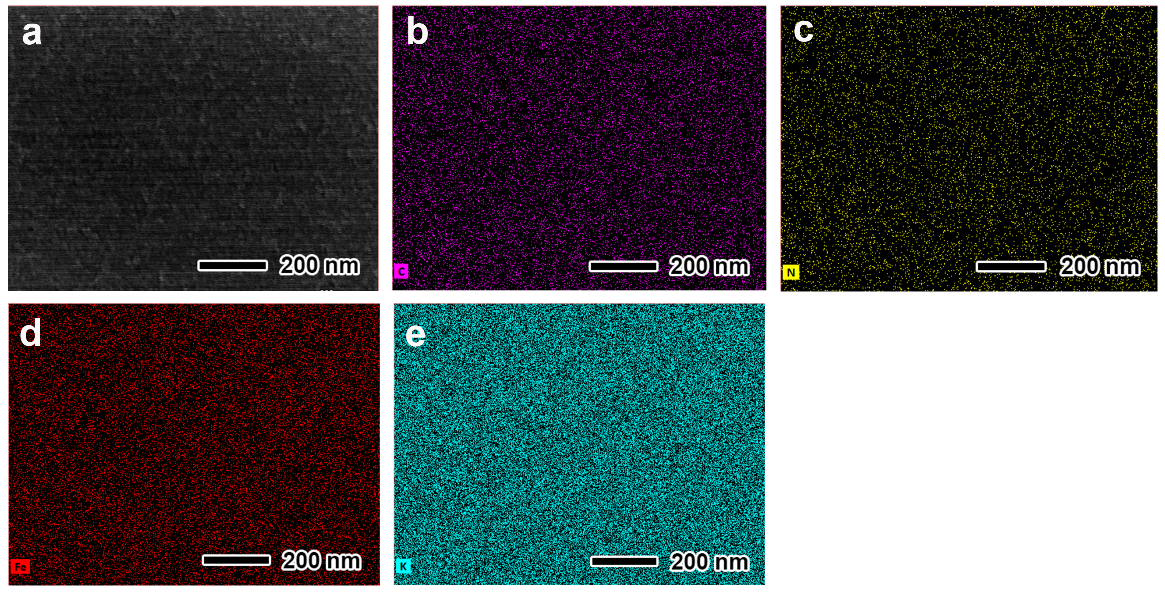


**Figure S6**. (a) The typical SEM image of the PB nanoparticle film and (b-e) the corresponding elemental distribution of C (b), N (c), Fe (d) and K (e).


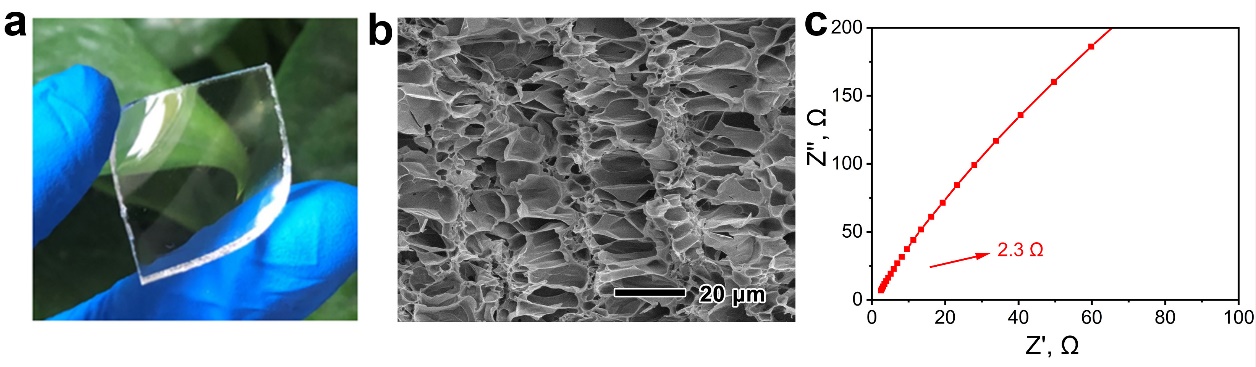


**Figure S7**. (a) Digital image of the PAM/LiCl gel film. (b) SEM image of the freeze-dried gel. (c) AC impedance spectra of the PAM/LiCl gel film.


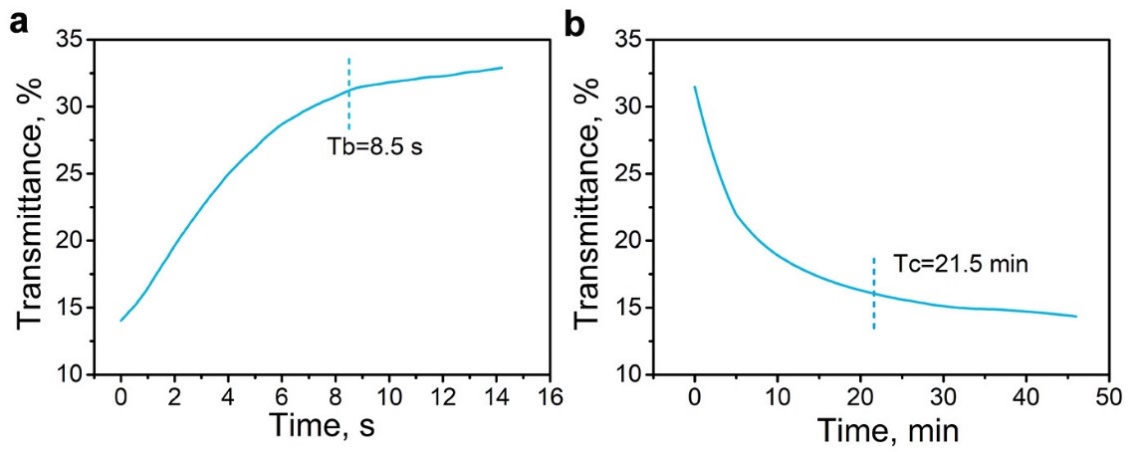


**Figure S8**. (a, b) Change of light transmittance at a wavelength of 400 nm of the NiHCF/gel film device versus time during bleaching (a) and coloration (b) processes.


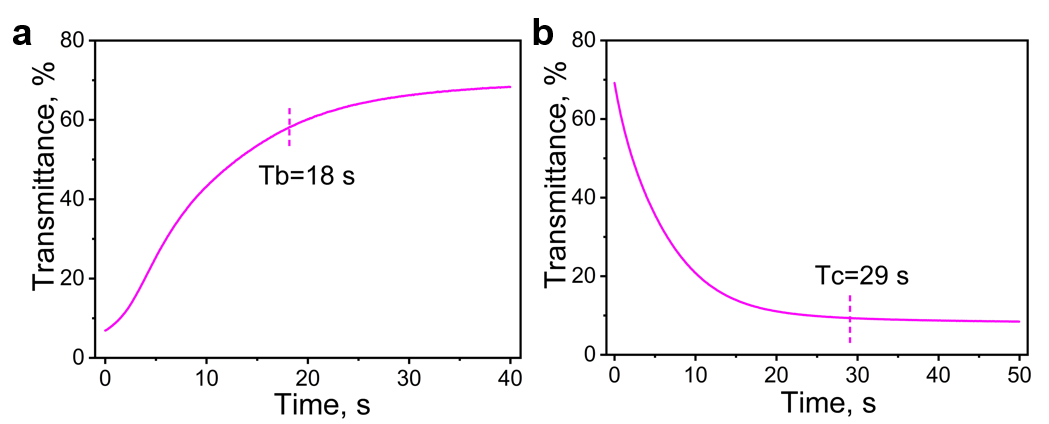


**Figure S9**. (a, b) Change of light transmittance at a wavelength of 700 nm of the PB/gel film device versus time during bleaching (a) and coloration (b) processes.


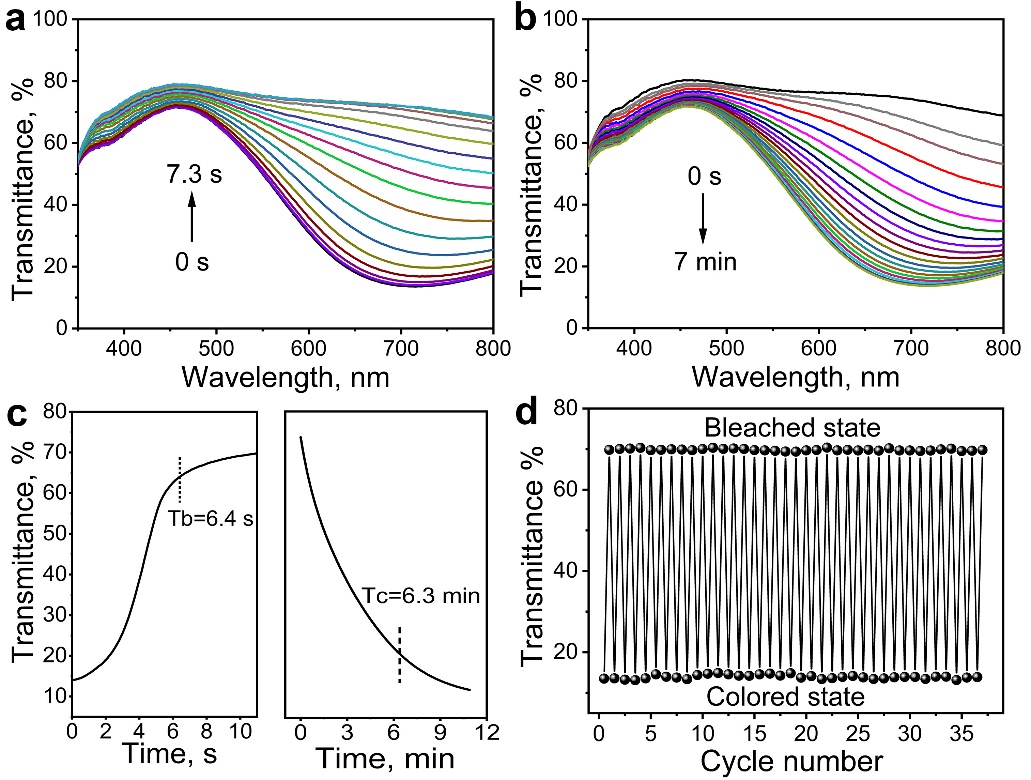


**Figure S10**. (a, b) UV-vis transmittance spectra showing bleaching process of PB/gel film device with 40 mg of (NH_4_)_2_S_2_O_8_ in the gel upon connecting the Al wire with PB nanoparticle film (a), and coloration process in air by disconnecting Al wire with PB nanoparticle film (b), respectively. (c) Change of light transmittance at a wavelength of 700 nm of the PB/gel film device versus time during bleaching (a) and coloration (b) processes. (d) The cycling property of the PB/gel film device.


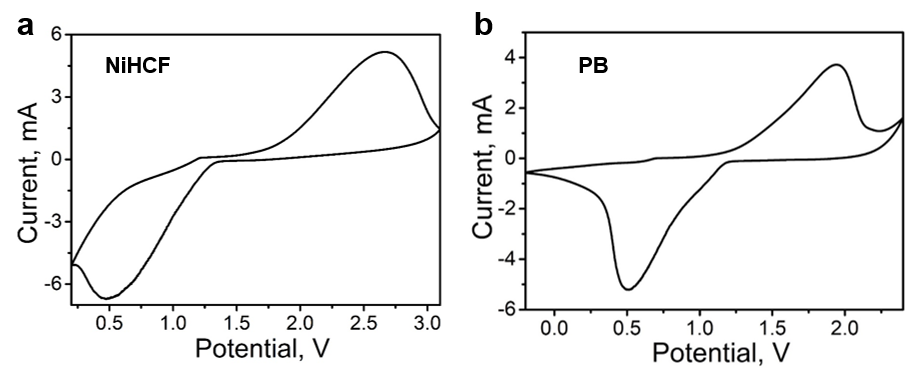


**Figure S11**. (a, b) The cyclic voltammogram of NiHCF/gel film device (a) and PB/gel film device (b) at a scan rate of 0.05 V s^-1^.


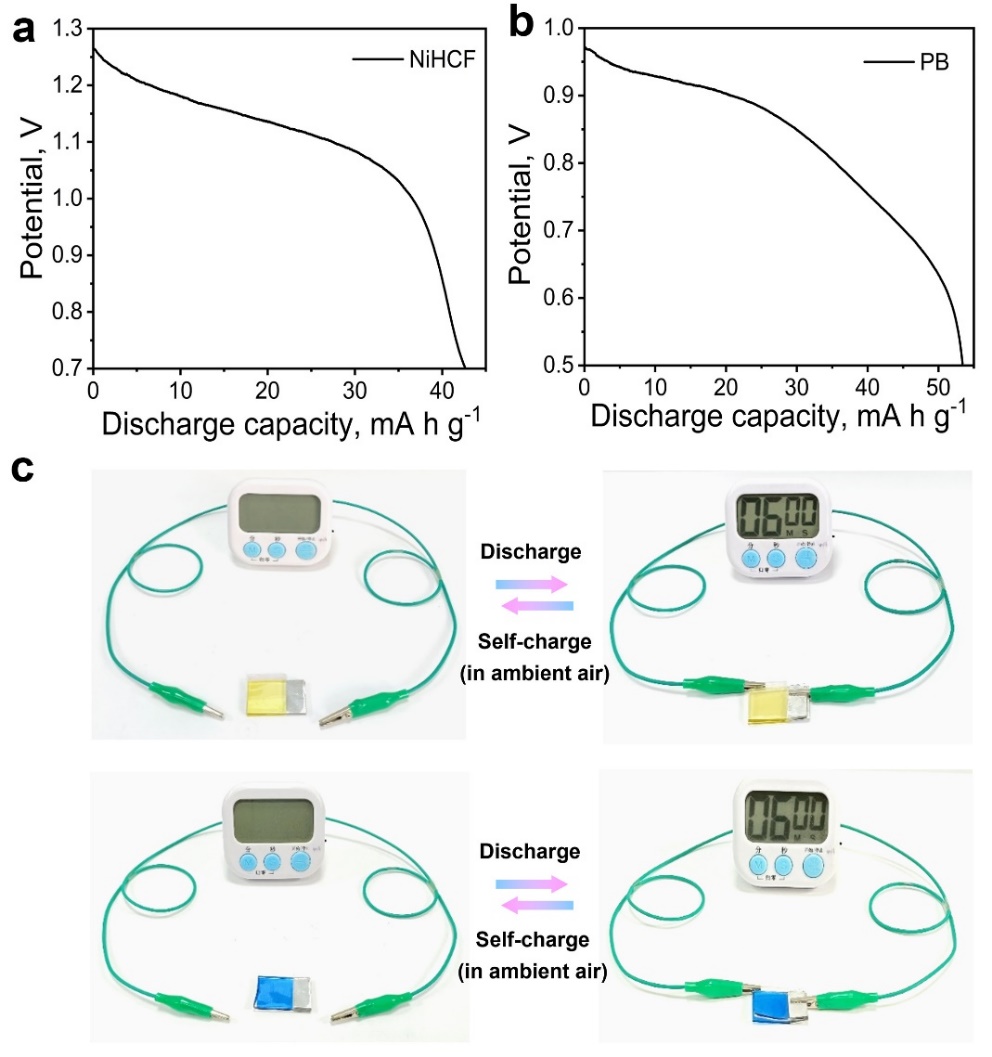


**Figure S12**. (a, b) Galvanostatic discharge curves of the NiHCF/gel film device (a) and PB/gel film device (b) at a current density of 2 A g^-1^. (c) Digital images of the NiHCF/gel film and PB/gel film acted as self-rechargeable batteries.


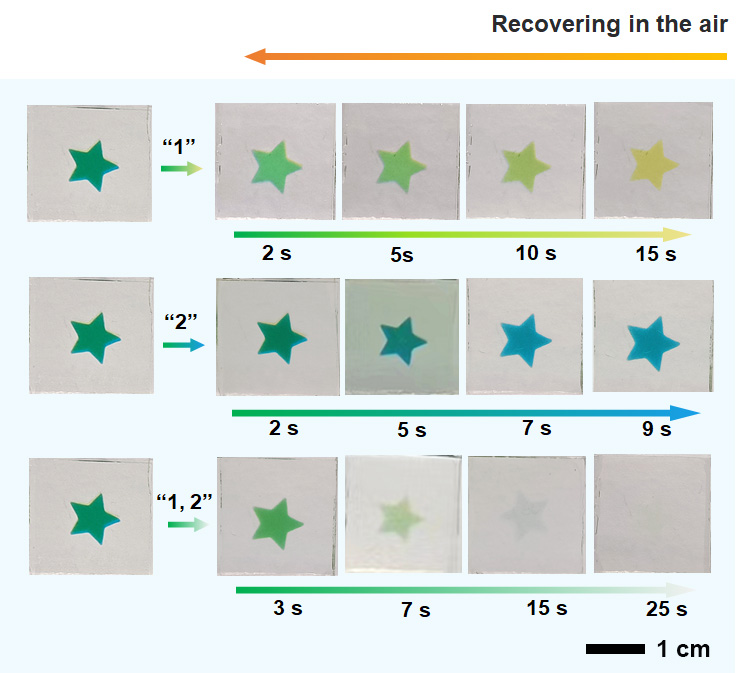


**Figure S13**. Digital images of the self-powered multicolor EC display with patterns showing color changing upon different connection sequence.

**Table S1** Comparison of our self-powered multicolor EC display with the electrochemical devices reported before.

| Ref | Electrochromic material | [Electrolyte](javascript:;) | Color | Bleaching time | Coloring time | Cycles | Flexible or not |
| --- | --- | --- | --- | --- | --- | --- | --- |
| [1] | PB | NaClO, KCl, phosphate buffer | monocolor | 50 s | 10 min | several cycles | not |
| [2] | PB | KCl solution | monocolor | 10 s | 4 h (45.8%) | - | not |
| [3] | PB | NaClO, KCl, phosphate buffer | monocolor | 10 s | 6.24 min | 7 | not |
| [4] | Polypyrrole | KCl solution | monocolor | 59.4 s | 1 h (48.3%) | - | not |
| [5] | PB | PAM/LiCl gel | monocolor | 6.8 s | 8 min | 50 | Yes |
| This work | PB | PAM/LiCl gel | monocolor | 19.2 s | 40 s | 55 | Yes |
|  | NiHCF |  | monocolor | 12.1 s | 30 min | 25 |  |
|  | PB/NiHCF |  | [multicolor](javascript:;) | 26 s | 60 min | 55 |  |

**References**

[1] Y. Zhai, Y. Li, H. Zhang, D. Yu, Z. Zhu, J. Sun, S. Dong, Self-rechargeable-battery-driven device for simultaneous electrochromic windows, ROS biosensing, and energy storage, *ACS Appl. Mater. Interfaces* vol. 11, no. 31, pp. 28072-28077, 2019.

[2] J. Wang, L. Zhang, L. Yu, Z. Jiao, H. Xie, X. W. Lou, X.W. Sun, A bi-functional device for self-powered electrochromic window and self-rechargeable transparent battery applications, *Nat. Commun.* vol. 5, article 4921, 2014.

[3] H. Zhang, Y. Yu, L. Zhang, Y. Zhai, S. Dong, Self-powered fluorescence display devices based on a fast self-charging/recharging battery (Mg/Prussian blue), *Chem. Sci.* vol. 7, no. 11, pp. 6721-6727, 2016.

[4] B. Yang, D. Ma, E. Zheng, J. Wang, A self-rechargeable electrochromic battery based on electrodeposited polypyrrole film, *Sol. Energy Mater. Sol. Cells* vol. 192, pp. 1-7, 2019.

[5] F. F. Zhao, J. M. Zhao, Y. Zhang, X. Wang, W. S. Wang, Self-powered quasi-solid-state electrochromic devices for optical information encryption, *J. Mater. Chem. C* vol. 9, no. 25, pp. 7958-7966, 2021.
